# Loop-Mediated Isothermal Amplification Screening for COVID-19 in Asymptomatic Preoperative Orthopedic Patients in a General Hospital in Kanagawa, Japan

**DOI:** 10.7759/cureus.9331

**Published:** 2020-07-22

**Authors:** Kentaro Uchida, Hiroyuki Sekiguchi, Reiji Higashiyama, Tachio Endo, Yuji Yokozeki, Gen Inoue, Masayuki Miyagi, Shotaro Fujino, Naoto Yoshida, Masashi Takaso

**Affiliations:** 1 Department of Orthopaedic Surgery, Kitasato University, School of Medicine, Sagamihara, JPN; 2 Medical Sciences Research Institute, Shonan University, Chigasaki, JPN; 3 Department of Orthopaedic Surgery, Shonantobu General Hospital, Chigasaki, JPN; 4 Orthopaedic Surgery, Chigasaki Chuo Hospital, Chigasaki, JPN; 5 Department of Orthopaedic Surgery, Kitasato University School of Medicine, Sagamihara, JPN; 6 Department of Orthopaedic Surgery, Shonantobu General Hospital, Sagamihara, JPN

**Keywords:** covid-19, orthopedic surgery

## Abstract

The global coronavirus disease 2019 (COVID-19) pandemic has caused several million infections and hundreds of thousands of deaths. A large number of healthcare workers have died as a result of infection with this virus. Therefore, elective surgery was markedly reduced or stopped in our hospital’s orthopedic department. The detection of asymptomatic COVID-19-positive patients became key to reducing the infection risk to physicians and staff to allow orthopedic surgery to be performed. A total of 21 patients were scheduled to undergo orthopedic surgery, including elective surgery, in Shonantobu General Hospital, Chigasaki City, Kanagawa, Japan. All 21 patients gave permission to undergo loop-mediated isothermal amplification (LAMP) screening the day before surgery. None of the 21 patients we tested was positive for COVID-19. All patients remained asymptomatic during the two to four weeks of postoperative follow-up. No physicians or medical staff developed COVID-19 symptoms. This was a very small study in a city with a relatively low incidence of COVID-19. We found that LAMP screening was accurate, in terms of its negative predictive value. Larger studies are needed.

## Introduction

The ﬁrst case of coronavirus disease 2019 (COVID-19) occurred in Wuhan, China, and was reported in December 2019. On January 16, 2020, the ﬁrst reports of COVID-19 were ofﬁcially announced by Japan’s Ministry of Health, Labour, and Welfare. The COVID-19 pandemic is the largest health care crisis of this century. A large number of health care workers have died following infection with this virus. As a consequence, elective surgery was markedly reduced or stopped in the orthopedic department of the hospital, despite its importance in preserving the quality of life and activities of daily living. A number of lines of evidence indicate that transmission of the virus by asymptomatic patients is possible and that this may have hastened the spread of COVID-19 [1-2]. Therefore, the management of asymptomatic patients to reduce the infection risk to physicians and medical staff is important to allow orthopedic surgery to be performed safely. Some guidelines for orthopedic procedures have been reported [3-5]. However, evidence-based clinical practices have not been fully developed.

Shonantobu General Hospital is located in Chigasaki City, Kanagawa Prefecture, Japan. Kanagawa Prefecture has the third-highest number of infected patients in Japan. The number of COVID-19-infected patients in Chigasaki City has been relatively small as compared to other cities in Kanagawa Prefecture. A strategy for the management of orthopedic surgery, including elective surgery, during the pandemic, was needed to maintain orthopedic surgery practice during and after this pandemic.

Loop-mediated isothermal amplification (LAMP) with simple visual detection of amplification provides rapid analysis in field applications [6]. LAMP was first established as a rapid and reliable method for amplifying a small amount of target sequence at a single reaction temperature, eliminating the requirement for sophisticated thermal cycling equipment. Previous studies have reported that the LAMP method is useful to detect COVID-19 [7]. We hypothesized that the LAMP method would be useful for screening for COVID-19 in asymptomatic patients undergoing orthopedic surgery.

## Materials and methods

Patients and methods

**Figure 1:**
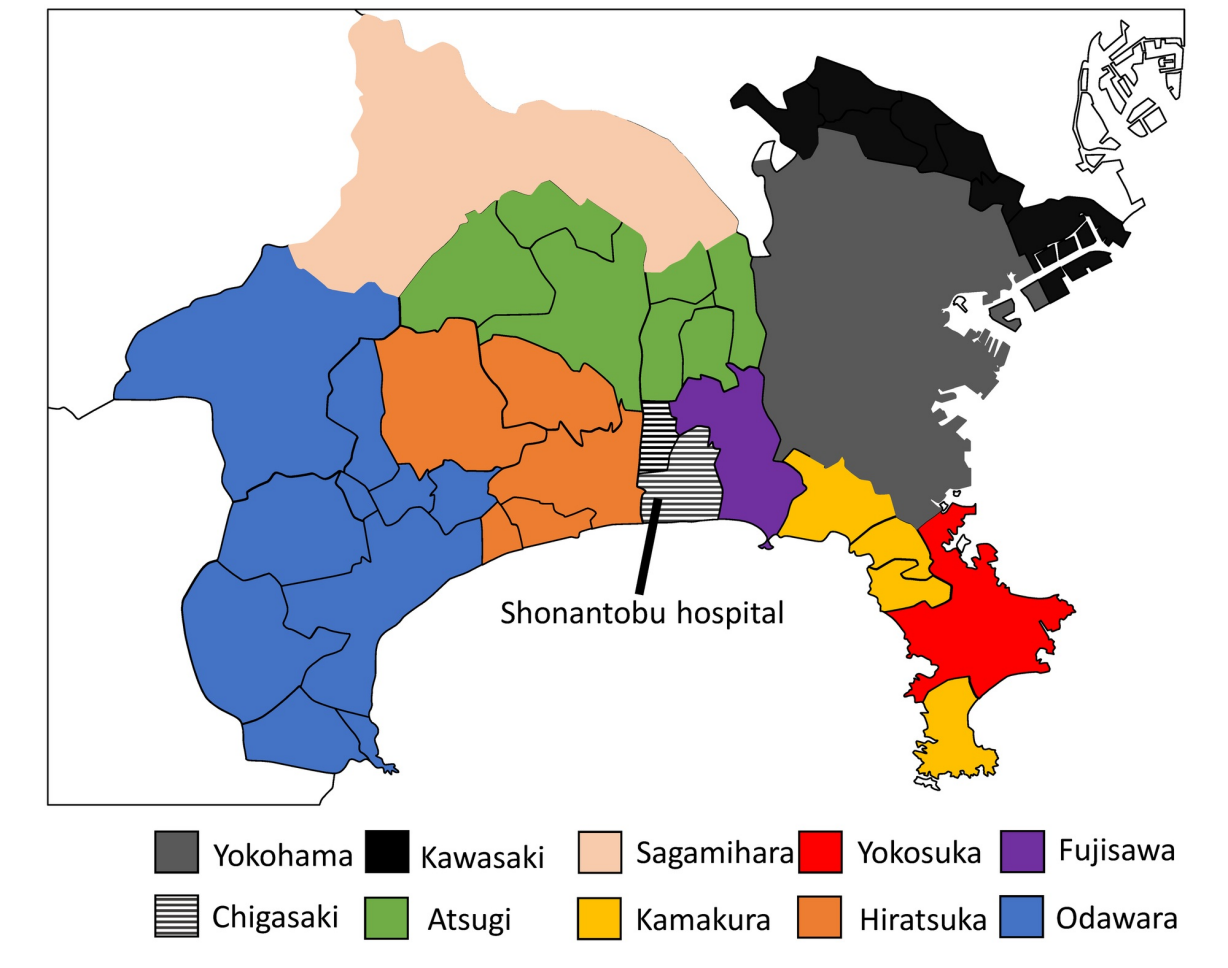
Map of Kanagawa Prefecture The number of COVID patients counted by Public Health Center in six cities, including Yokohama, Kawasaki, Sagamihara, Yokosuka, Chigasaki, and Fujisawa City, and by the public health and welfare office, which examined four cities, including Kamakura, Atsugi, Hiratsuka, and Odawara. We divided these into 10 areas.

Syonantobu Hospital is located in Chigasaki City in the south-central Kanagawa Prefecture (Figure 1).

The number of COVID patients were counted by Public Health Centers at six cities: Yokohama, Kawasaki, Sagamihara, Yokosuka, Chigasaki, and Fujisawa City and by public health and welfare offices at four cities, including Kamakura, Atsugi, Hiratsuka, and Odawara. We estimated COVID-19 patient numbers in 10 areas of Kanagawa Prefecture from February 12 to May 24, 2020, using information from the Kanagawa Prefecture official home page [8]. We also calculated the approximate incidence of COVID cases in populations in each area.

This study was approved by the Shonantobu Institutional Review Board (approval number 2020-005). Informed consent was obtained from all individual participants. A total of 21 patients (age 21-90 y) without symptoms of fever, shortness of breath, or desaturation were included. Patients underwent LAMP screening using a commercial kit (Loopamp®, Eiken Chemical Co., Ltd, Tokyo, Japan) for COVID-19 detection one day before surgery. Nasopharyngeal samples were used to detect COVID-19. Total viral ribonucleic acid (RNA) was extracted from the specimens using the RNA extraction kit (QIAamp Viral RNA Mini Kit, Qiagen, Hilden, Germany) according to the manufacturer's instructions. Isolated RNA reacted with enzyme mix (avian myeloblastosis virus (AMV) reverse transcriptase and Bst DNA polymerase) and primer mix at 62.5°C for 35 min according to the manufacture’s protocol. Turbidity measurement was carried out at an optical density of 650 nm, and the reaction was considered positive when the turbidity values were >0.1.

## Results

Epidemiology

**Figure 2:**
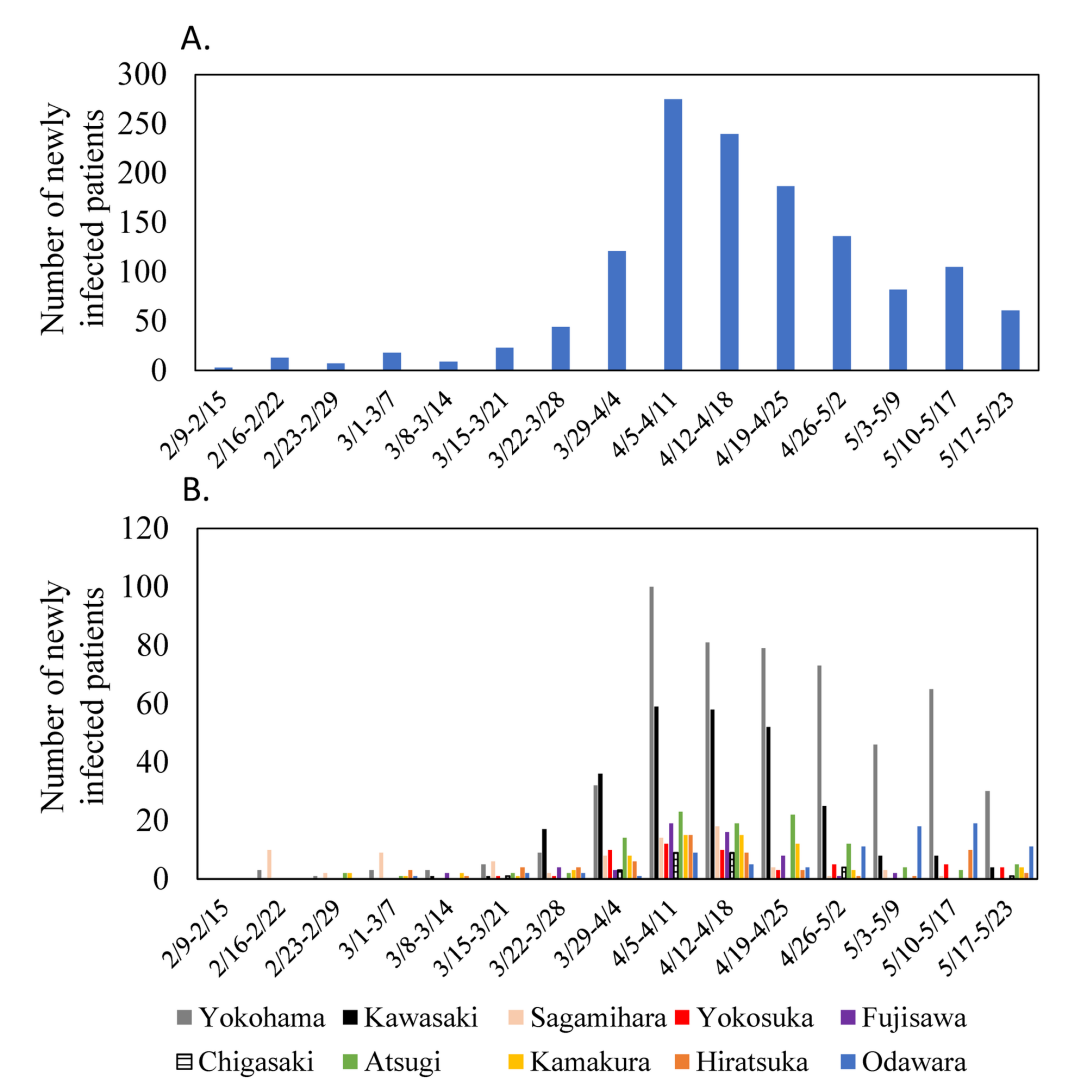
Sequential changes in the number of COVID-19 patients in Kanagawa Prefecture and 10 areas A. COVID-19 patients in Kanagawa Prefecture, B. COVID-19 patients in 10 areas

In Japan, the weekly rate of infection gradually increased from February 2-9 to April 5-11. Prime Minister Shinzo Abe declared a state of emergency in seven urban areas, including Kanagawa Prefecture, on April 7 extending until May 25. After the declaration of the state of emergency, the number of newly infected patients gradually decreased in Kanagawa Prefecture (Figures 2A-2B).

Most cases in Kanagawa Prefecture were concentrated in Yokohama and Kawasaki Cities (Table 1; 530 and 269 patients, respectively). Twenty-seven cases were observed in Chigasaki City; this rate was lower than that in the other nine areas (Table 1).

**Table 1:** COVID patients in Kanagawa Prefecture cities 1. Chigasaki Public Health Center calculated the patients’ numbers in Chigasaki and Samukawacho. 2. Atsugi public health and welfare office calculated the patients’ numbers in Atsugi, Ebina, Zama, Aikawa, Kiyokawa, Yamoto, and Ayase. Therefore, the number of patients and the population in the Atsugi area included these cities and towns. 3. Kamakura public health and welfare office calculated the patients’ numbers in Kamakura, Zushi, Miura, and Hayama. Therefore, the number of patients and population in Kamakura included these cities and towns. 4. The Hiratsuka public health and welfare office calculated the patient’s number in Hiratsuka, HadanoIsehara, Oiso, and Ninomiyamachi. Therefore, the number of patients and the population in Hiratsuka included these cities and towns. 5. The Odawara public health and welfare office calculated the patient numbers in Odawara City, Minamiashigara, Nakaimachi, Oimachi, Matsudamachi, Yamakitamachi, Oimachi, Matsudamachi, Yamakitamachi, Kaiseimachi, Hakone, Yamakitamachi, and Manazuru. Therefore, the number of patients and the population in the Odawara area included these cities and towns.

City	Population	COVID patients	COVID cases/billion people
Yokohama	3,753,771	530	1.41
Kawasaki	1,535,415	269	1.75
Sagamihara	722,252	78	1.08
Yokosuka	390,549	51	1.31
Fujisawa	435,121	55	1.26
Chigasaki^1^	290,411	27	0.93
Atsugi^2^	856,148	109	1.27
Kamakura^3^	304,150	62	2.04
Hiratsuka area^4^	583,073	59	1.01
Odawara^5^	319,641	83	2.60

Incidence of COVID-19 in patients scheduled to undergo orthopedic surgery

None of the 21 patients we tested was positive for COVID-19. All underwent orthopedic surgery. Nine patients requiring fracture repair and one patient with a spinal tumor underwent emergency surgery. Eleven cases, including one each with osteoarthritis, ligament tear, and meniscus tear, underwent elective surgery following negative COVID-19 screening. The clinical characteristics of the patients are listed in Table 2.

**Table 2:** Clinical characteristics of patients undergoing orthopedic surgery Arthroscopic surgery, AS; Diabetes mellitus, DM; Hypertension, HT; HTO, High tibial osteotomy; Lumbar spinal stenosis, LSS; OA, osteoarthritis; ORIF, open reduction and internal fixation; TKA, Total knee arthroplasty THA, Total hip arthroplasty; PLF, Posterior lumbar fusion

	Date	Sex, age (y)	Orthopedic diagnosis	Complication	Surgery	Elective/emergency
Case 1	Apr 30	F, 77	Hip OA	HT, DM	THA	Elective
Case 2	Apr 30	M, 54	Elbow fracture	None	ORIF	Emergency
Case 3	May 1	M, 76	metastatic spinal tumor	Lung cancer	PLF	Emergency
Case 4	May 1	F, 86	Hip OA	HT	THA	Elective
Case 5	May 2	F, 61	Calcaneal fracture	HT, Hepatitis C	ORIF	Emergency
Case 6	May 2	F, 82	LSS	None	Decompression surgery	Elective
Case 7	May 8	M, 35	Meniscus tear	None	AS	Elective
Case 8	May 8	F, 49	Knee OA	HT	HTO	Elective
Case 9	May 13	F, 68	Femoral neck fracture	HT	THA	Emergency
Case 10	May 13	M, 28	Diaphysealradius fracture	None	ORIF	Emergency
Case 11	May 14	M, 54	Lateral humeral epicondylitis	HT, DM	AS	Elective
Case 12	May 15	M, 37	Subtalar joint pseudarthrosis	hyperuricemia	AS	Elective
Case 13	May 15	M, 38	Ankle ligament tear	None	AS	Elective
Case 14	May 16	F, 88	Femoral Trochanteric fracture	None	ORIF	Emergency
Case 15	May 20	F, 90	Femoral Trochanteric fracture	HT	ORIF	Emergency
Case 16	May 20	F, 52	Ankle fracture	None	External fixation	Emergency
Case 17	May 20	F, 63	Fifth finger proximal phalanx fracture	None	Percutaneous wire fixation	Emergency
Case 18	May 21	F, 64	Knee OA	None	TKA	Elective
Case 19	May 21	M, 46	Clavicle fracture	None	ORIF	Emergency
Case 20	May 22	M, 38	Ankle ligament tear	None	AS	Elective
Case 21	May 22	F, 21	Ankle ligament tear	None	AS	Elective

All patients remained asymptomatic after orthopedic surgery during the two to four weeks follow-up. No physicians or medical staff developed symptoms of COVID-19.

## Discussion

The global COVID-19 pandemic is caused by the SARS-CoV-2 virus and has so caused several million infections, with hundreds of thousands of deaths around the world. Previous studies have reported on emergency orthopedic surgery during a pandemic. A recent study reported that the occurrence of COVID-19 pneumonia in patients with a fracture can result in severe adverse outcomes and increase mortality [9]. Surgery was performed following pneumonia and COVID-19 testing under pandemic conditions and significantly improved outcomes. Upper extremity numbness was resolved in patients with a spinal tumor [10]. In contrast, the widespread recommendation to postpone elective operations in the US during the pandemic has produced a large population of patients with hip and knee osteoarthritis who are unable to receive their recommended surgical treatment [11]. In our cases, we performed both emergency and elective surgery following COVID-19 screening with LAMP during a declared state of emergency in Japan. No complications were observed after surgery in any patient. During the study period, 1324 COVID-19 cases were reported in Kanagawa Prefecture. However, COVID-19 cases in Chigasaki City, where our hospital is located, were lower than in other cities. Our experience with LAMP screening may provide a strategy to begin orthopedic surgery, including elective surgery, in asymptomatic patients with orthopedic problems in a post-pandemic future.

A previous study found the colorimetric LAMP assay provided 100% agreement with quantitative reverse transcription-polymerase chain reaction (RT-qPCR) results across a range of Cq values and rapidly detects COVID-19 [7]. Here, we used the LAMP method as a rapid detection tool to test asymptomatic patients with orthopedic problems. A LAMP assay screening may be important to ensure the safety of the medical staff and other patients.

Some studies have reported that concerns have been raised about the poor sensitivity of nucleic acid-based tests. The sensitivity of PCR was this might be as low as 59% [12]. On the day of symptom onset, the median false-negative rate was 38% [13]. Therefore, further investigations with larger sample sizes are needed to clarify the usefulness of the LAMP method to identify a COVID-19 infection in patients requiring orthopedic surgery in the post-pandemic future.

## Conclusions

A total of 21 patients gave permission to undergo LAMP screening the day before orthopedic surgery. None of the patients we tested was positive for COVID-19. All patients remained asymptomatic during the two to four weeks of postoperative follow-up. No physicians or medical staff developed COVID-19 symptoms. LAMP screening may provide a strategy to begin orthopedic surgery, including elective surgery, in asymptomatic patients with orthopedic problems in a post-pandemic future.

## References

[1] Bai Y, Yao L, Wei T, Tian F, Jin DY, Chen L, Wang M (2020). Presumed asymptomatic carrier transmission of COVID-19. JAMA.

[2] Li C, Ji F, Wang L (2020). Asymptomatic and human-to-human transmission of SARS-CoV-2 in a 2-family cluster, Xuzhou, China. Emerg Infect Dis.

[3] Hirschmann MT, Hart A, Henckel J, Sadoghi P, Seil R, Mouton C (2020). COVID-19 coronavirus: recommended personal protective equipment for the orthopaedic and trauma surgeon. Knee Surg Sports Traumatol Arthrosc.

[4] Mouton C, Hirschmann MT, Ollivier M, Seil R, Menetrey J (2020). COVID-19 - ESSKA guidelines and recommendations for resuming elective surgery. J Exp Orthop.

[5] Sarac NJ, Sarac BA, Schoenbrunner AR (2020). A review of state guidelines for elective orthopaedic procedures during the COVID-19 outbreak. J Bone Joint Surg Am.

[6] Notomi T, Okayama H, Masubuchi H, Yonekawa T, Watanabe K, Amino N, Hase T (2000). Loop-mediated isothermal amplification of DNA. Nucleic Acids Res.

[7] Baek YH, Um J, Antigua KJC (2020). Development of a reverse transcription-loop-mediated isothermal amplification as a rapid early-detection method for novel SARS-CoV-2. Emerg Microbes Infect.

[8] (2020). Kanagawa Prefecture official homepage [In Japanese]. https://www.pref.kanagawa.jp/docs/ga4/bukanshi/occurrence_03.html.

[9] Mi B, Chen L, Xiong Y, Xue H, Zhou W, Liu G (2020). Characteristics and early prognosis of COVID-19 infection in fracture patients. J Bone Joint Surg Am.

[10] Kessler RA, Zimering J, Gilligan J (2020). Neurosurgical management of brain and spine tumors in the COVID-19 era: an institutional experience from the epicenter of the pandemic. J Neurooncol.

[11] Brown TS, Bedard NA, Rojas EO (2020). The effect of the COVID-19 pandemic on electively scheduled hip and knee arthroplasty patients in the United States. J Arthroplasty.

[12] Ai T, Yang Z, Hou H (2020). Correlation of chest CT and RT-PCR testing in coronavirus disease 2019 (COVID-19) in China: a report of 1014 cases. Radiology.

[13] Kucirka LM, Lauer SA, Laeyendecker O, Boon D, Lessler J (2020). Variation in false-negative rate of reverse transcriptase polymerase chain Reaction-based SARS-CoV-2 tests by time since exposure. Ann Intern Med.

